# Serum Adiponectin and In Vivo Brain Amyloid Deposition in Cognitively Normal Older Adults: A Cohort Study

**DOI:** 10.14336/AD.2022.1118

**Published:** 2023-06-01

**Authors:** Jee Wook Kim, Min Soo Byun, Dahyun Yi, Min Jung Kim, Joon Hyung Jung, Nayeong Kong, Gijung Jung, Hyejin Ahn, Jun-Young Lee, Koung Mi Kang, Chul-Ho Sohn, Yun-Sang Lee, Yu Kyeong Kim, Dong Young Lee, for the KBASE Research Group

**Affiliations:** ^1^Department of Neuropsychiatry, Hallym University Dongtan Sacred Heart Hospital, 7 Keunjaebong-gil, Hwaseong, Gyeonggi, Korea.; ^2^Department of Psychiatry, Hallym University College of Medicine, Chuncheon, Gangwon, Korea.; ^3^Department of Neuropsychiatry, Seoul National University Hospital, Seoul, Korea.; ^4^Department of Psychiatry, Seoul National University College of Medicine, Seoul, Korea.; ^5^Institute of Human Behavioral Medicine, Medical Research Center Seoul National University, Seoul, Korea.; ^6^Department of Psychiatry, Eulji University Nowon Eulji Medical Center, Seoul, Korea.; ^7^Department of Neuropsychiatry, SMG-SNU Boramae Medical Center, Seoul, Korea.; ^8^Department of Radiology, Seoul National University Hospital, Seoul, Korea.; ^9^Department of Nuclear Medicine, Seoul National University College of Medicine, Seoul, Korea.; ^10^Department of Nuclear Medicine, SMG-SNU Boramae Medical Center, Seoul, Korea

**Keywords:** adiponectin, Aβ, Alzheimer’s disease;, cognitive normal, tau, neurodegeneration

## Abstract

High blood adiponectin has been associated with Alzheimer’s disease (AD) dementia and related cognitive decline. We aimed to investigate the association between serum adiponectin level and *in vivo* AD pathologies. Cross-sectional and longitudinal study designs for the data of the Korean Brain Aging Study for Early Diagnosis and Prediction of Alzheimer’s Disease, an ongoing prospective cohort study that began in 2014. A total of 283 cognitively normal older adults between 55 and 90 years of age were included in community and memory clinic setting. Participants underwent comprehensive clinical assessments, measurement of serum adiponectin level, and multimodal brain imaging, including Pittsburgh compound-B positron emission tomography (PET), AV-1451 PET, fluorodeoxyglucose (FDG)-PET, and MRI at baseline and 2-year follow-up. Serum adiponectin level was positively associated with global beta-amyloid protein (Aβ) retention and change therein over 2 years, but not with other AD neuroimaging markers including tau deposition, AD-related neurodegeneration, and white matter hyperintensities. Blood adiponectin level is associated with increased brain amyloid deposition, which suggests that adiponectin may be a potential target for therapeutic and preventive strategies against AD.

## INTRODUCTION

Increasing evidence suggests that Alzheimer’s disease (AD), the most common cause of dementia, is a brain metabolic disorder which is intrinsically linked to adipose tissue dysfunction and adipokines-mediated signals [1-6]. From this point of views on linking AD and dysfunctional adipose tissue and adipokines, adiponectin has been proposed as a new clue to understand the pathogenesis of AD [4, 7-11]. Adiponectin [12], a collagen-like 30 kDa protein hormone, is the most abundant adipokine in circulation, representing 0.01% of the total plasma proteins in humans [13]. Adiponectin is primarily released from adipose tissue into the bloodstream^1^ and involved in numerous metabolic pathways including glucose flux, lipid catabolism, insulin sensitivity, and energy metabolism as well as inflammatory processes [14-16]. Many human studies have shown cross-sectional [17-21] and longitudinal association [22] between high blood adiponectin level and clinically defined AD dementia or late-life cognitive decline. A study showed that individuals with AD dementia and mild cognitive impairment (MCI) showed higher blood adiponectin than cognitively normal (CN) individuals [20]. Such finding for the cross-sectional association between high blood adiponectin and AD dementia was confirmed by other studies [17-19, 21]. A population-based longitudinal study[22] also showed that high blood adiponectin level was an independent risk factor for the development of AD dementia.

Nevertheless, the neuropathological links underlying the association between high blood adiponectin level and AD dementia have not yet been clearly elucidated. Although there were a couple of human studies on the pathological links, the findings were conflicting. The Mayo Clinic Study of Aging (MCSA) [23] for non-demented older adults reported that high serum adiponectin was related with decreased hippocampal volume in female with elevated brain beta-amyloid protein (Aβ) deposition. In contrast, another human study [24] showed a positive association between serum adiponectin level and hippocampal volume. The studies had significant limitations in that they had cross-sectional study design and did not measure brain tau deposition, one of the core AD brain pathologies. In addition, the latter study only included 45 diabetic men aged 50 and 75 years, which limits the generalizability of study results.

In this context, we examined the association between serum adiponectin level and *in vivo* AD pathologies, including cerebral Aβ and tau deposition, and AD-related regional neurodegeneration, as measured on multi-modal brain imaging, in CN older adults using both cross-sectional and longitudinal approaches. We also investigated the association between serum adiponectin level and white matter hyperintensities (WMHs) on MRI as a measure of cerebrovascular white matter injury.

## METHODS

### Participants

This study is part of the Korean Brain Aging Study for Early Diagnosis and Prediction of Alzheimer’s Disease (KBASE), which is an ongoing prospective cohort study that began in 2014 [25]. As of October 2019, a total of 283 CN participants between 55 and 90 years of age were enrolled in the study.

Participants were recruited through four recruitment sites around Seoul, South Korea. Potentially eligible individuals who participated in a dementia screening program at two public centers for dementia prevention and management or visited memory clinics at two university hospitals [i.e., Seoul National University Hospital (SNUH) and Seoul National University-Seoul Metropolitan Government (SNU-SMG) Boramae Medical Center] around Seoul, South Korea, were informed about study participation and those who volunteered were invited for an assessment of eligibility. In addition, volunteers from the community were recruited through advertisements at an online homepage, posters, and brochures provided at main recruitment sites and word of mouth (recommended by other participants, family members, friends, or acquaintances).

The CN group consisted of participants with a Clinical Dementia Rating (CDR) score of 0 and no diagnosis of mild cognitive impairment (MCI) or dementia. The exclusion criteria were the following: 1) presence of a major psychiatric illness; 2) significant neurological or medical condition or comorbidity that could affect mental functioning; 3) contraindications for an MRI scan (*e.g.*, pacemaker or claustrophobia); 4) illiteracy; 5) presence of significant visual/hearing difficulties and/or severe communication or behavioral problems that would make clinical examinations or brain scans difficult; 6) pregnancy or lactation; and, 7) use of an investigational drug.

### Standard protocol approvals, registrations, and participants consent

This study protocol was approved by the institutional review boards of the Seoul National University Hospital (C-1401-027-547) and the Seoul Metropolitan Government-Seoul National University (SMG-SNU) Boramae Medical Center (26-2015-60), in Seoul, South Korea; and we conducted it in accordance with the recommendations of the current version of the Declaration of Helsinki. All the participants gave written informed consent.

### Clinical assessments

Participants received standardized clinical and neuropsychological assessments at baseline by trained board-certified psychiatrists. The Consortium to Establish a Registry for Alzheimer’s Disease (CERAD) clinical and neuropsychological assessment battery is one of the well-established, globally used standard clinical assessment protocol to evaluate AD and related cognitive impairment state (https://agingcenter.duke.edu/cerad/) [26, 27]. The assessments were based on the Korean Brain Aging Study for the KBASE clinical assessment protocol, which incorporated the Korean version of the Consortium to Establish a Registry for Alzheimer’s Disease (CERAD-K) clinical assessment [25, 28, 29]. A clinical neuropsychologist or trained psychometrists also applied a comprehensive neuropsychological assessment battery to the participants, following a standardized protocol incorporating the CERAD-K neuropsychological battery. Details on the full assessment battery have been previous described [25-29].

### Measurement of serum adiponectin level and blood tests for potential confounders

After an overnight fast, blood samples were obtained via venipuncture in the morning (8-9 a.m.) at baseline. Serum adiponectin was measured with the EZHADP-61K human adiponectin enzyme-linked immunosorbent assays (ELISA) kit (Merck Millipore, Darmstadt, Germany). We also measured serum albumin, total cholesterol, HDL- and LDL-cholesterol, fasting glucose, insulin, and zinc levels, which may possibly confound the association between adiponectin and brain pathologies [23, 30-32]. Serum albumin, total cholesterol, HDL- and LDL-cholesterol, and glucose levels were measured using a colorimetry method (ADVIA 1800 Auto Analyzer, Siemens, Washington, DC, USA). Fasting insulin was measured with chemiluminescent immunoassay method (ADVIOA Centaur XP, Siemens, Washington, DC, USA). We measured serum zinc levels using an inductively coupled plasma-mass spectrometer (model 820-MS; Bruker, Australia). Homeostasis model assessment (HOMA) was used to estimate insulin resistance (IR). HOMA-IR was calculated with the formula: fasting plasma glucose (mmol/L) times fasting serum insulin (mU/L) divided by 22.5 [33]. We categorized the HOMA-IR status into two groups based on the evidence that HOMA-IR value above 2.9 indicates significant IR[34, 35]. Additionally, genomic DNA was extracted from whole blood and apolipoprotein E (APOE) genotyping was performed as described [36]. APOE ε4 (APOE4) positivity was defined as the presence of at least one ε4 allele.

### Assessment of other potential confounders

The association between adiponectin and AD pathology may be influenced by various factors. Serum adiponectin is highly sensitive to age, sex, physical activity, annual income, depression, vascular risk, body mass index (BMI), alcohol drinking, and, smoking [23, 30, 31, 37, 38] as well as various blood markers mentioned above [23, 30-32]. As all the factors are related to AD or the risk of AD, they have potential to confound or moderate the association between adiponectin and AD pathologies. Therefore, we systematically evaluated those factors for all participants. The detailed procedures of evaluations for the potential confounders other than blood markers were described as follows. We assessed physical activity using the interviewer-administered Lifetime Total Physical Activity Questionnaire, a tool with demonstrated reliability [39, 40] and validity [41]. This questionnaire assesses occupational, household, and leisure activities separately throughout a respondent’s lifetime. We assessed the frequency and duration of these activities by recording the number of years, months per year, weeks per month, days per week and hours per day that each activity was performed. The intensity of activity was estimated by the participants as sedentary, light, moderate or heavy. A metabolic equivalent (MET) value was assigned to each activity based on the Compendium of Physical Activities [42]. We calculated LPA scores as the sum of the MET-h/week spent on occupational, household, and leisure activities over the lifetime, and we categorized the LPA status into two groups based on the median quartile (high LPA group *vs.* low LPA group). We also assessed annual incomes and categorized them into three groups (below the minimum cost of living [MCL], more than MCL but below two MCLs, and two or more MCLs (www.law.go.kr). The MCL was determined according to the administrative rule published by the Ministry of Health and Welfare, Republic of Korea, in November 2012. The MCL is 572,168 Korea Won (KRW) for single-person household and adds 286,840 KRW for each additional housemate. We used the Geriatric Depression Scale (GDS)[43, 44] to measure the severity of depressive symptoms [43]. The comorbidity rates of vascular risk factors were assessed by interviewing the participants and their reliable informants; we calculated a vascular risk score (VRS) based on the number of vascular risk factors present and reported the VRS as a percentage [45]. The BMI was calculated using the weight in kilograms divided by the height in meters squared and we categorized participants into three groups (<18.4 as underweight, 18.5 to 24.9 as normal weight, 25 as overweight to obese) according to the World Health Organization (WHO) guideline (https://apps.who.int/iris/bitstream/handle/10665/63854/WHO_NUT_NCD_98.1_%28p159276%29.pdf). We assessed the average lifetime alcohol intake status (never/former/drinker), and average lifetime smoking status (never/ex-smoker/smoker) after interviewing the participants. To acquire accurate information, reliable informants were interviewed, and medical records were reviewed.

### Measurement of cerebral Aβ deposition

Participants underwent simultaneous three-dimensional (3D) [^11^C] Pittsburg compound B (PiB)-positron emission tomography (PET) and 3D T1-weighted MRI scans using a 3.0T Biograph mMR (PET-MR) scanner (Siemens; Washington DC, WC, USA) at baseline and 2-year follow-up visit. The details of PiB-PET acquisition and preprocessing were previously described [36]. An automatic anatomic labeling algorithm [46] and a region combining method [47] were applied to determine regions of interests (ROIs) to characterize the PiB retention level in the frontal, lateral parietal, posterior cingulate-precuneus, and lateral temporal regions. A global Aβ retention value was the mean standardized uptake value ratio (SUVR) for all voxels of the four ROIs, calculated by dividing the mean uptake value of a reference region. For cross-sectional analysis of baseline data, the inferior cerebellar gray matter in the Spatially Unbiased Infratentorial Template for the cerebellum (SUIT) atlas [48] was used as the reference region for intensity normalization. For longitudinal analysis, the reference region included the inferior cerebellar grey matter in the SUIT, cerebellar white matter (thresholded at 50%) [49], pons, and cerebrum white matter (thresholded at 95% and eroded by 3 voxels) [50, 51].

### Measurement of cerebral tau deposition

A subset of participants (n = 73) underwent [^18^F] AV-1451 PET scans (Siemens) using a Biograph True point 40 PET/CT scanner (Siemens), in accordance with the manufacturer’s guidelines. While all the other neuroimaging scans were performed at baseline and then at 2-year follow visit, the AV-1451 PET imaging was initially conducted at an average of 2.5 (standard deviation 0.3) years delayed time point from the baseline visit and then at 2 years after the initial scan. The details of AV-1451 PET imaging acquisition and preprocessing have been described [36]. To estimate cerebral tau deposition we quantified the AV-1541 SUVR of an a priori ROI of “AD-signature regions” of tau accumulation, which comprised a size-weighted average of partial volume-corrected uptake in entorhinal, amygdala, parahippocampal, fusiform, inferior temporal, and middle temporal ROIs. We used the AV-1541 SUVR of the above-mentioned ROI as an outcome variable for cerebral tau deposition [36]. For cross-sectional analysis of baseline data, ^18^F-AV-1451 PET SUVR images based on the mean uptake over 80 to 100 min post-injection, normalized by the mean inferior cerebellar grey matter uptake (based to the code published by Baker *et al.*[52]). For longitudinal analysis, literature recommends using cerebrum white matter as the reference region for intensity normalization [53]. Using the abovementioned code, the reference region was changed to hemispheric white ROI from FreeSurfer instead of inferior cerebellar gray ROI in the partial volume correction code [52].

### Measurement of AD-related neurodegeneration

All participants underwent [^18^F] fluorodeoxyglucose (FDG)-PET imaging using the above-described PET-MR machine at baseline and 2-year follow-up visit. The details of FDG-PET image acquisition and preprocessing were previously described [54]. Voxel-weighted mean SUVR was extracted from the AD-signature FDG ROIs known to be sensitive to metabolic changes associated with AD [55, 56], which include the angular gyri, posterior cingulate cortex, and inferior temporal gyri. AD-signature region cerebral glucose metabolism (AD-CM) was defined as a voxel-weighted mean SUVR of the AD-signature FDG ROIs. For measurement of AD-signature region cortical thickness (AD-CT), all T1 MR images were obtained using the PET-MR machine mentioned above. The details of MRI acquisition and preprocessing were previously described [54]. The AD-CT was calculated as the average cortical thickness of the AD-signature regions, including the entorhinal, inferior temporal, middle temporal, and fusiform gyri [55].

### Measurement of WMHs

All participants underwent MRI scans with fluid-attenuated inversion recovery using the abovementioned PET-MR scanner. We followed the validated automatic procedure reported previously with some minor modification [57, 58]. Briefly, the procedure consisted of 11 steps: spatial co-registration of T1 and FLAIR images, fusion of T1 and FLAIR images, T1 segmentation, attainment of transformation parameters, deformation and obtainment of the white matter mask, obtainment of FLAIR within the white matter mask, intensity normalization of the masked FLAIR, nomination of candidate WMH with a designated threshold, creation of a junction map, and elimination of the junction. Using the final WMH candidate image, the WMH volume was extracted in the native space for each participant.

### Statistical analysis

To examine the association between serum adiponectin and baseline or longitudinal changes in brain pathologies, we performed multiple linear regression with serum adiponectin level as an independent variable and corresponding baseline or longitudinal changes in brain neuroimaging measures including Aβ and tau deposition, AD-CM, or AD-CT, and WMH as dependent variable. In the analyses, global Aβ retention was used after natural log-transformation to achieve normal distribution. The change in neuroimaging measures for each participant were calculated as the difference between the follow-up and the baseline values, which were represented as delta (Δ). Three models were tested for stepwise control of potential confounders that could affect the relationships between serum adiponectin level and biomarkers. The first model did not include any covariate. The second model included age, sex, and APOE4 as covariates. The third model included all potential covariates, including age, sex, APOE4, education, GDS score, VRS, BMI, annual income, LPA, albumin, glucose, HDL-/LDL-cholesterol, zinc, alcohol intake, smoking, and HOMA-IR, which have been considered as possible confounders in previous studies [23, 30-32, 37, 38]. As sensitivity analyses, the same analyses were performed only for well-nourished participants (i.e., serum albumin ≥ 3.5 g/dL and total cholesterol ≥ 150 mg/dL)[59, 60] to exclude the effect of malnourishment on adiponectin level [61, 62].

In addition, we explored the moderation effects of age, sex, APOE4, education, GDS, VRS, BMI status, annual income, LPA status, HDL-and LDL-cholesterol, zinc, alcohol, smoking, and HOMA-IR status on the relationships between serum adiponectin level and the neuroimaging biomarker(s), which showed a significant association with adiponectin in above analyses: We included two-way interaction term between adiponectin and any one of the factors, as well as adiponectin itself, as an independent variable in the regression model. For significant interaction(s), subsequent subgroup analyses were performed using an additional regression model for each subgroup divided by the moderation variable. All statistical analyses were performed using SPSS Statistics software (version 27; IBM Corp., Armonk, NY, USA).

**Figure 1.**
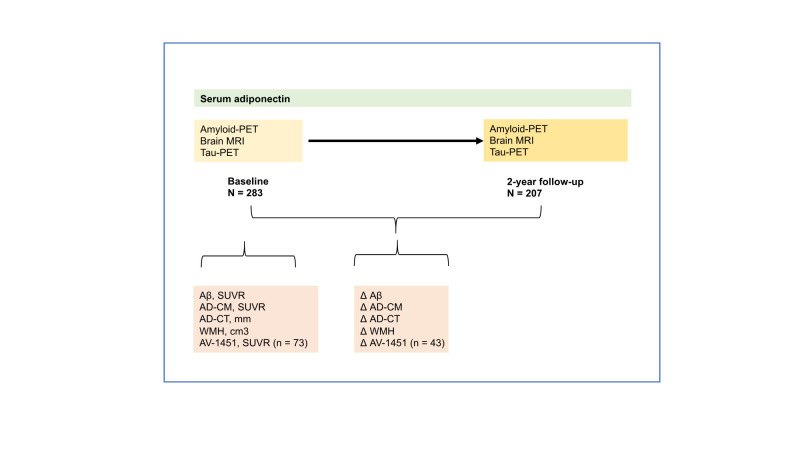
An outline of the study design and analyses. CN = cognitively normal, MRI = Magnetic Resonance Imaging, PET = Positron Emission Tomography, AD-CM = AD-signature region cerebral glucose metabolism, AD-CT = AD-signature regional cortical thickness, WMH = white matter hyperintensity.

## RESULTS

### Participant characteristics

Table 1 presents the characteristics of the study participants. Among the 283 CN participants who underwent baseline evaluations, 207 (73.1%) completed the second neuroimaging assessments at the 2-year follow-up. Figure 1 presents an outline of the study design and cross-sectional and longitudinal analyses for the associations of serum adiponectin with neuroimaging markers and changes therein over 2 years.

**Table 1:** Participant characteristics.

Characteristics	Baseline	2-year follow-up
N	283	207
Age, y	69.18 (8.07)	
Female, n (%)	147 (51.94)	
Education, y	11.80 (4.82)	
MMSE	26.93 (2.48)	
APOE4 positivity, n (%)	52 (18.37)	
Clinical diagnosis, CN, n (%)	283 (100.00)	
Lifetime physical activity, MET-h/week	77.79 (56.04)	
Annual income, n (%)		
<MCL	20 (7.07)	
≥MCL, <2×MCL	119 (42.05)	
≥2×MCL	144 (50.88)	
Vascular risk score, %	17.37 (16.05)	
Geriatric depression scale	4.77 (4.98)	
Alcohol intake status, n (%)		
Never/Former/Drinker	143 (50.53)/35 (12.37)/105 (37.10)	
Smoking status, n (%)		
Never/Former/Smoker	183 (64.66)/83 (29.33)/17 (6.01)	
Body mass index, kg/m^2^		
Underweight (<18.5)	4 (1.41)	
Normal weight (18.5-24.9)	172 (60.78)	
Overweight (≥25)	107 (37.81)	
Serum adiponectin, ng/ml	10,370 (6,063.26)	
Nutritional markers		
Albumin	4.48 (0.24)	
Glucose, fasting	104.74 (20.65)	
HDL-Cholesterol	53.57 (14.07)	
LDL-Cholesterol	108.51 (30.10)	
Insulin	8.03 (4.52)	
HOMA-IR	2.10 (1.29)	
High	62 (21.91)	
Low	221 (78.09)	
Zinc	86.92 (11.85)	
Malnutrition, n (%)	44 (15.55)	
Cerebral Aβ deposition		
Aβ retention, SUVR	1.19 (0.22)	1.22 (0.29)
Aβ retention, SUVR		<0.01 (0.05)
Cerebral tau deposition		
AV-1451, SUVR (n = 73, 43)	1.33 (0.24)	1.38 (0.23)
ΔAV-1451, SUVR		0.04 (0.16)
Neurodegeneration		
AD-CM, SUVR	1.42 (0.12)	1.33 (0.15)
ΔAD-CM, SUVR		-0.09 (0.15)
AD-CT, mm	2.87 (0.17)	2.81 (0.16)
ΔAD-CT, mm		-0.05 (0.14)
WMH volume, cm^3^	12.87 (13.09)	13.94 (14.93)
ΔWMH volume, cm^3^		0.51 (12.18)

APOE4=apolipoprotein ε4, CN=cognitively normal, MCL=minimum cost of living, Aβ=beta-amyloid, AD=Alzheimer’s disease, AD-CM=Alzheimer’s disease signature cerebral glucose metabolism, AD-CT=Alzheimer’s disease signature cortical thickness, SUVR standardized uptake value ratio, WMH white matter hyperintensities. Unless otherwise indicated, data are expressed as means (standard deviations).

### Cross-sectional association between adiponectin and in vivo brain pathological markers

**Figure 2.**
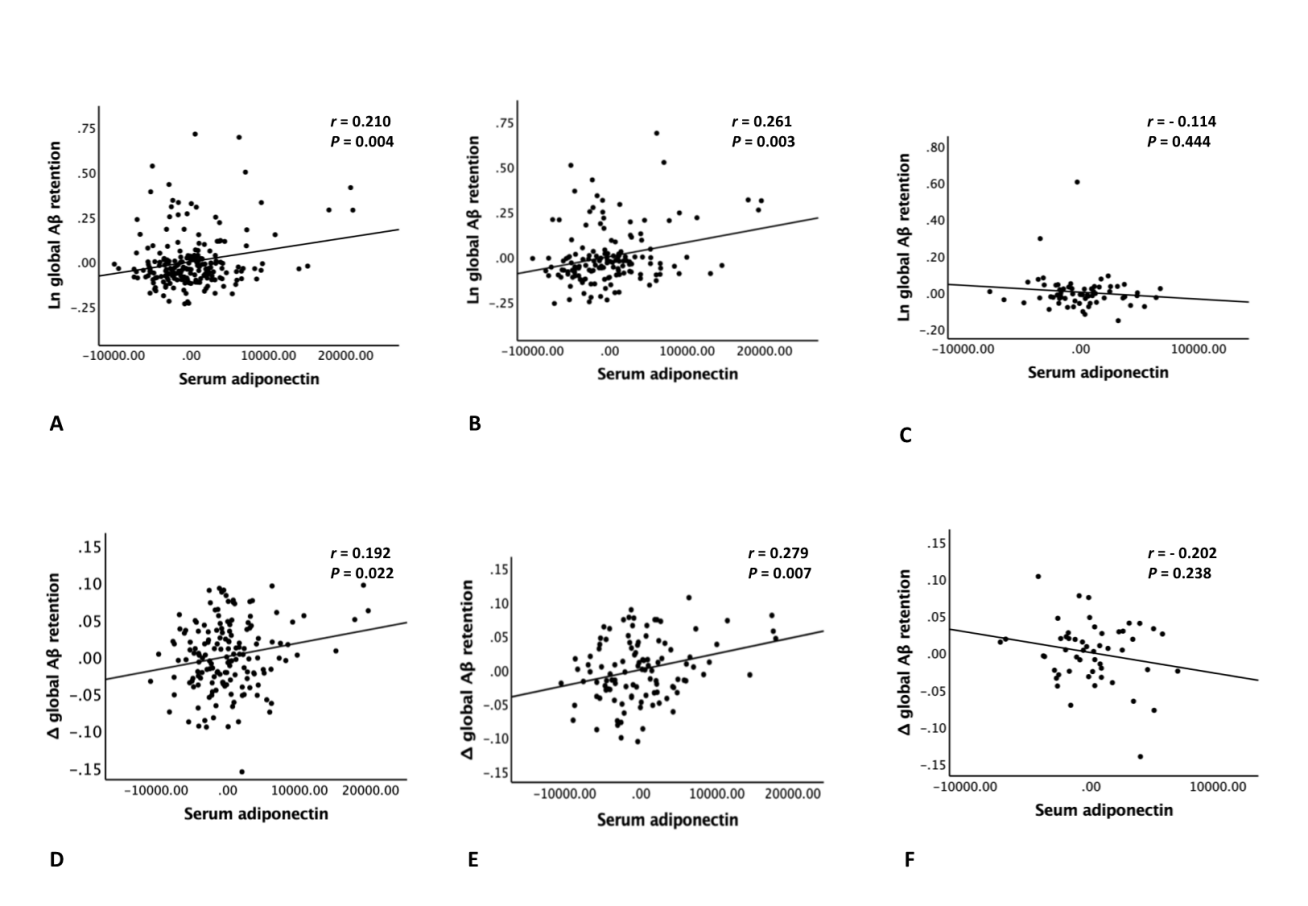
Plots of association between serum adiponectin level and global Aβ retention. Plots of the cross-sectional (A-C) and longitudinal (D-F) associations of serum adiponectin level with global Aβ retention (A, D) in overall group (n = 283, 207), in (B, E) older (aged ≥ 65 years), and (C, F) younger (aged < 65 years) subgroups (n = 191, 137, 92, 70). *Aβ* = beta-amyloid. Multiple linear regression analyses were performed after adjusting for all potential covariates.

**Figure 3.**
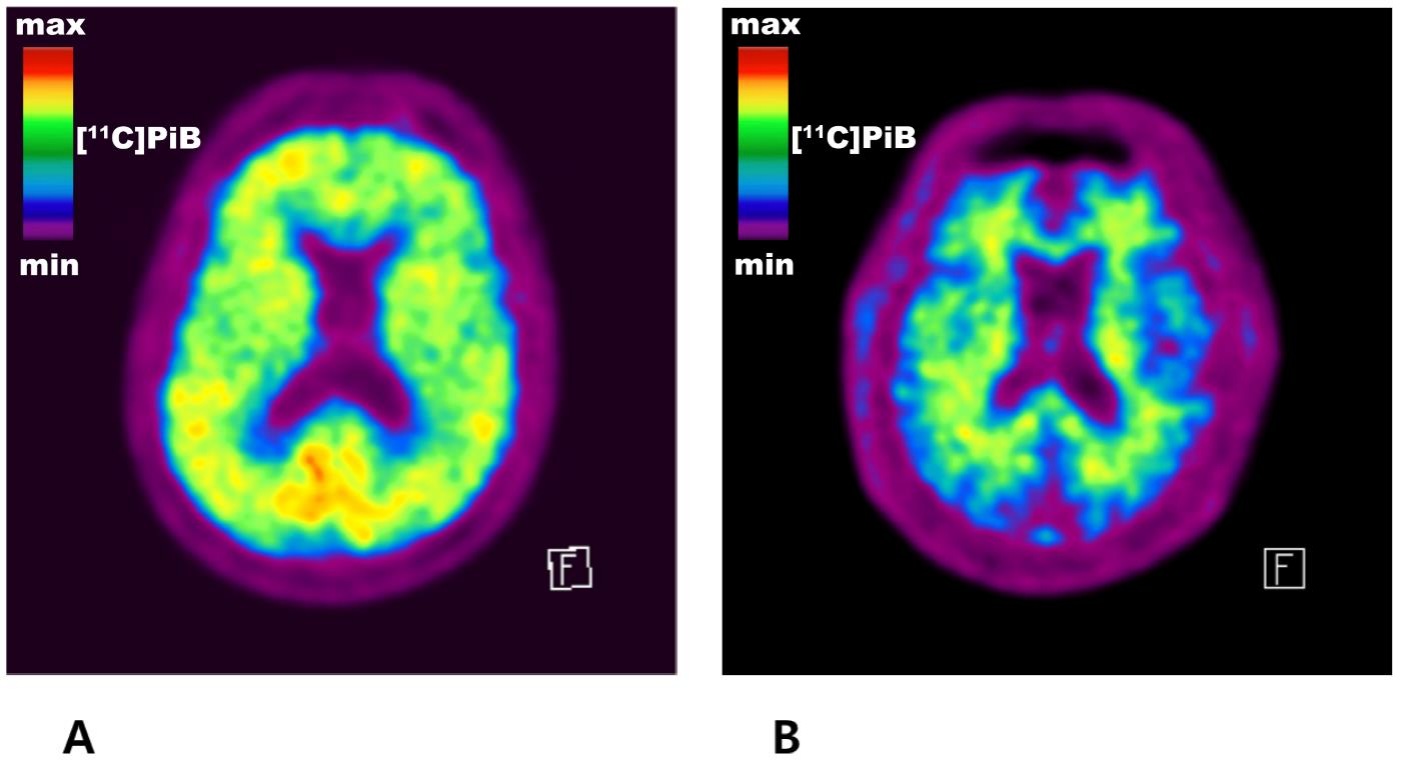
Representative images of carbon 11-labeled Pittsburgh compound B positron emission tomography ([^11^C] PiB-PET). (A) An individual (male, 74 years old) with high serum adiponectin level (21,491 ng/ml) and a typical amyloid positive scan of [^11^C] PiB-PET showing beta-amyloid (Aβ) retention in the cerebral gray matter. (B) An individual (male, 74 years old) with low serum adiponectin level (2,683 ng/ml) and a typical amyloid negative scan of [^11^C] PiB-PET showing only nonspecific uptake in the cerebral white matter and no Aβ uptake in the gray matter.

Table 2 and Figure 2A present the cross-sectional association between serum adiponectin level and global Aβ retention. Serum adiponectin level was positively associated with global A retention, regardless of the models tested. Representative images of [^11^C] PiB-PET from an individual with high serum adiponectin level and an individual with low serum adiponectin level are also provided in Figure 3. In contrast, there was no significant association between serum adiponectin level and tau deposition, AD-CM, AD-CT, and WMH volume (Table 3). Sensitivity analyses only for the nourished participants showed similar results (Tables 4 and 5).

### Longitudinal association between adiponectin and in vivo brain pathological markers

Similar to the cross-sectional findings, baseline serum adiponectin level was positively associated with global Aβ changes over 2 years after adjustment for the potential covariates (Table 2 and Fig. 2D), but not with the changes in other neuroimaging markers (Table 3).

**Table 2:** Results of cross-sectional and longitudinal analyses of the associations between serum adiponectin and global Aβ retention in cognitively normal individuals.

	Global Aβ retention, SUVR		Δ global Aβ retention, SUVR	
	β	*p-value*	β	*p-value*
Independent variable: adiponectin				
Model 1^a^	0.276	<0.001^***^	0.171	0.031^*^
Model 2^b^	0.230	<0.001^***^	0.168	0.038^*^
Model 3^c^	0.248	0.004^**^	0.230	0.022^*^

Aβ=beta-amyloid, =standardized beta coefficients.

a Not adjusted.

b Adjusted for age, sex, and APOE4.

c Adjusted for age, sex, APOE 4, education, GDS score, VRS, BMI, annual income, LPA, albumin, fasting glucose, HDL- and LDL-cholesterol, HOMA-IR, zinc, alcohol intake, and smoking.

* *p* < 0.05,

** *p* < 0.01,

*** *p* < 0.001.

### Moderation effects of potential confounders on the association between adiponectin and in vivo brain Aβ deposition and longitudinal changes therein

We also explored the moderation effects of age, sex, APOE4, education, GDS, VRS, BMI status, annual income, LPA status, HDL-and LDL-cholesterol, zinc, alcohol, smoking, and HOMA-IR on the associations of serum adiponectin with global Aβ deposition and 2-year changes thein. There was significant interaction between adiponectin level and age, indicating that age moderates the association of adiponectin with baseline and Δ values of Aβ retention (Table 6). The subgroup analyses showed that adiponectin level was positively associated with Aβ deposition and 2-year changes thein in older (aged 65 years and older) individuals, but not in younger (aged less than 65 years of age) individuals (Tables 7 and 8, and Figure 2B, C, E, and F). In contrast, no significant interactions were noted between adiponectin level and other factors (Table 6).

**Table 3:** Results of cross-sectional and longitudinal analyses of the associations between serum adiponectin and AV-1451, AD-CM, AD-CT, or WMH volume in cognitively normal individuals.

	AV-1451, SUVR		AD-CM, SUVR		AD-CT, mm		WMH volume, cm^3^	
	β	*p-value*	β	*p-value*	β	*p-value*	β	*p-value*
Model 1^a^	0.227	0.087	-0.070	0.314	-0.145	0.040*	0.051	0.473
Model 2^b^	0.144	0.286	-0.018	0.802	-0.039	0.553	0.017	0.811
Model 3^c^	0.132	0.553	-0.079	0.361	-0.023	0.777	0.027	0.771
	ΔAV-1451, SUVR		ΔAD-CM, SUVR		ΔAD-CT, mm		ΔWMH volume, cm^3^	
	β	*p-value*	β	*p-value*	β	*p-value*	β	*p-value*
Model 1^a^	0.171	0.326	0.074	0.350	0.076	0.353	-0.058	0.478
Model 2^b^	0.289	0.095	0.016	0.837	0.077	0.369	-0.101	0.219
Model 3^c^	0.066	0.789	0.053	0.560	0.106	0.319	-0.125	0.235

Aβ=beta-amyloid, AD-CM=Alzheimer’s disease signature cerebral glucose metabolism, AD-CT=Alzheimer’s disease signature cortical thickness, WMH=white matter hyperintensity, =standardized beta coefficients.

a Not adjusted.

b Adjusted for age, sex, and APOE4.

c Adjusted for age, sex, APOE 4, education, GDS score, VRS, BMI, annual income, LPA, albumin, fasting glucose, HDL- and LDL-cholesterol, HOMA-IR, zinc, alcohol intake, and smoking.

* *p* < 0.05,

** *p* < 0.01,

*** *p* < 0.001.

**Table 4:** Results of cross-sectional and longitudinal analyses of the associations between adiponectin and Aβ deposition in cognitively normal individuals without malnutrition.

	Global Aβ retention, SUVR		Δ global Aβ retention, SUVR	
	*β*	*p-value*	*β*	*p-value*
Independent variable: adiponectin				
Model 1^a^	0.321	<0.001^***^	0.219	0.010 ^*^
Model 2^b^	0.266	<0.001^***^	0.201	0.023^*^
Model 3^c^	0.310	<0.001^***^	0.294	0.007^**^

Aβ=beta-amyloid, =standardized beta coefficients.

a Not adjusted.

b Adjusted for age, sex, and APOE4.

c Adjusted for age, sex, APOE 4, education, GDS score, VRS, BMI, annual income, LPA, albumin, fasting glucose, HDL- and LDL-cholesterol, HOMA-IR, zinc, alcohol intake, and smoking.

* *p* < 0.05,

** *p* < 0.01,

*** *p* < 0.001.

## DISCUSSION

This cross-sectional and longitudinal study revealed that serum adiponectin level was significantly associated with increased Aβ deposition in CN older adults, while it was not associated with other AD pathologies or cerebrovascular injury. Our finding of the positive association between serum adiponectin level and Aβ deposition is in line with the results of previous studies that reported a relationship between high serum adiponectin level and increased risk of AD dementia and late-life [17-22].

**Table 5:** Results of cross-sectional and longitudinal analyses of the associations between serum adiponectin and AV-1451, AD-CM, AD-CT, or WMH volume in cognitively normal individuals without malnutrition.

	AV-1451, SUVR		AD-CM, SUVR		AD-CT, mm		WMH volume, cm^3^	
	β	*p-value*	β	*p-value*	β	*p-value*	β	*p-value*
Model 1^a^	0.256	0.076	-0.105	0.105	-0.175	0.022^*^	0.065	0.396
Model 2^b^	0.184	0.237	-0.068	0.387	-0.056	0.426	0.028	0.728
Model 3^c^	0.137	0.615	-0.115	0.226	-0.010	0.907	0.042	0.671
	ΔAV-1451, SUVR		ΔAD-CM, SUVR		ΔAD-CT, mm		ΔWMH volume, cm^3^	
	β	*p-value*	β	*p-value*	β	*p-value*	β	*p-value*
Model 1^a^	0.114	0.535	0.088	0.297	0.102	0.249	-0.056	0.523
Model 2^b^	0.264	0.166	0.036	0.670	0.115	0.215	-0.107	0.233
Model 3^c^	0.164	0.508	0.070	0.480	0.100	0.389	-0.150	0.189

Aβ=beta-amyloid, AD-CM=Alzheimer’s disease signature cerebral glucose metabolism. AD-CT=Alzheimer’s disease signature cortical thickness. WMH=white matter hyperintensity, =standardized beta coefficients.

a Not adjusted.

b Adjusted for age, sex, and APOE4.

c Adjusted for age, sex, APOE 4, education, GDS score, VRS, BMI, annual income, LPA, albumin, fasting glucose, HDL- and LDL-cholesterol, HOMA-IR, zinc, alcohol intake, and smoking.

* *p* < 0.05,

** *p* < 0.01,

*** *p* < 0.001.

The exact mechanism underlying the association between serum adiponectin level and brain Aβ deposition is not clear. In humans, low blood levels of adiponectin are related to the development of type 2 diabetes and metabolic syndrome, which have been epidemiologically linked to increased risk of AD dementia [63]. Therefore, the positive relationship of high blood adiponectin level with increased risk of AD dementia or higher brain Aβ deposition cannot not be explained by the direct insulin-sensitizing, anti-diabetic, or anti-metabolic-syndrome properties of adiponectin [64]. Although the evidence is insufficient, other novel mechanisms may be involved. Some evidence suggests that the compensatory adaption by adiponectin to insulin resistance state may be involved in the AD pathogenesis [19]. Given that the network of insulin/IGF-1 receptor signaling pathway is essential in brain [65], upregulation of blood adiponectin may reflect a compensatory feedback response to abnormally reduced insulin/IGF-1 receptor signaling due to insulin resistance [66]. Because the integrity of the blood-brain barrier (BBB) gradually decreases with aging [67, 68], increased adiponectin in blood may leak into the brain parenchyma and contribute to the sequestration of amyloidogenic proteins [19]. In addition to the adaptive increase of blood adiponectin level, insulin resistance or compromised insulin receptor signaling pathway may downregulate expression of insulin degrading enzyme (IDE), a downstream target of the pathway and protease that digests Aβ as well as insulin [69]. As a result, decreased brain IDE activity can lead to accelerated Aβ accumulation. Nevertheless, in our study, the relationship between serum adiponectin level and brain Aβ deposition remained significant even after controlling for HOMA-IR (Model 3); therefore, insulin resistance-related events may not be the sole mechanism underlying the relationship.

**Table 6:** Cross-sectional and longitudinal moderation effects of age, sex, APOE4, education, GDS, VRS, BMI, annual income, lifetime physical activity, albumin, fasting glucose, HDL-and LDL-cholesterol, and HOMA-IR on association between serum adiponectin and global Aβ retention in cognitively normal individuals.

	Global Aβ retention, SUVR		Δ global Aβ retention, SUVR	
	*β*	*p-value*	*β*	*p-value*
Adiponectin	-0.314	0.074	-0.301	0.139
Age	0.047	0.510	-0.012	0.896
Adiponectin×Age	0.580	0.002^**^	0.468	0.030^*^
Adiponectin	0.119	0.212	0.084	0.382
Sex	-0.090	0.484	0.015	0.912
Adiponectin×Sex	0.223	0.104	0.115	0.410
Adiponectin	0.125	0.066	0.092	0.274
APOE4	-0.060	0.575	-0.045	0.727
Adiponectin×APOE4	0.213	0.065	0.144	0.308
Adiponectin	-0.062	0.717	0.076	0.670
Education	-0.037	0.779	0.160	0.246
Adiponectin×Education	0.333	0.092	0.074	0.717
Adiponectin	0.213	0.014	0.102	0.328
GDS	0.058	0.624	0.013	0.927
Adiponectin×GDS	-0.059	0.658	0.073	0.659
Adiponectin	0.226	0.011	0.081	0.456
VRS	0.013	0.911	-0.061	0.673
Adiponectin×VRS	-0.100	0.422	0.124	0.418
Adiponectin	0.492	0.085	0.732	0.029
BMI	0.146	0.229	0.198	0.174
Adiponectin×BMI	-0.252	0.370	-0.578	0.079
Adiponectin	0.199	0.207	0.061	0.749
Annual income	0.071	0.543	0.012	0.931
Adiponectin×Annual income	-0.020	0.914	0.096	0.667
Adiponectin	0.331	0.118	0.133	0.617
Lifetime physical activity	0.044	0.718	0.002	0.989
Adiponectin×Lifetime physical activity	-0.147	0.538	-0.001	0.997
Adiponectin	-0.324	0.796	-1.389	0.368
Albumin	-0.043	0.731	-0.112	0.473
Adiponectin×Albumin	0.506	0.683	1.514	0.321
Adiponectin	0.618	0.065	0.132	0.777
Fasting glucose	0.136	0.230	0.016	0.917
Adiponectin×Fasting glucose	-0.423	0.192	0.010	0.982
Adiponectin	-0.056	0.814	-0.096	0.730
HDL-cholesterol	-0.172	0.129	-0.200	0.137
Adiponectin×HDL-cholesterol	0.350	0.228	0.360	0.292
Adiponectin	0.129	0.579	0.249	0.460
LDL-cholesterol	-0.018	0.877	0.016	0.913
Adiponectin×LDL-cholesterol	0.064	0.800	-0.123	0.735
Adiponectin	0.489	0.105	-0.096	0.793
HOMA-IR	-0.228	0.432	-0.012	0.948
Adiponectin×HOMA-IR	-0.228	0.432	0.302	0.391
Adiponectin	0.514	0.307	-0.492	0.430
Zinc	0.030	0.818	-0.107	0.524
Adiponectin×Zinc	-0.312	0.533	0.668	0.283
Adiponectin	0.147	0.054	0.161	0.084
Alcohol	-0.210	0.084	-0.054	0.714
Adiponectin×Alcohol	0.108	0.370	-0.057	0.700
Adiponectin	0.170	0.012	0.090	0.275
Smoking	-0.174	0.156	-0.156	0.319
Adiponectin×Smoking	0.039	0.731	0.164	0.258

APOE4=apolipoprotein ε4, GDS=geriatric depression scale, BMI=body mass index, Aβ=beta-amyloid, =standardized beta coefficients. The moderation effects of potential covariate on the relationships between adiponectin and Aβ retention were examined by multiple linear regression analyses including two-way interaction term between adiponectin and any one of the confounders, as well as adiponectin itself, as an independent variable.

* *p* < 0.05,

** *p* < 0.01,

*** *p* < 0.001.

The detrimental effect of adiponectin on Aβ deposition was more prominent in older (aged ≥ 65 years) compared to younger (aged < 65 years) individuals, which may be related to age-related impairment of the BBB integrity mentioned previously. Increased blood adiponectin is more likely to leak into the brain parenchyma of older individuals with relatively damaged BBB compared to younger individuals with relatively preserved BBB. This finding may also be due in part to age-related difference in Aβ deposition in CN individuals [70]. As shown in Table 7, older individuals had a higher mean level and greater variation in Aβ deposition compared to younger individuals at both baseline and 2-year follow-up time point. This difference in Aβ level and distribution may explain the discrepancy between age subgroups. In contrast to age, other factors did not have a moderation effect on the association between adiponectin level and Aβ deposition.

**Table 7:** Aβ deposition in older and younger individuals.

	Older (≥65 years)	Younger (< 65 years)	t or χ^2^	*p-value*
Age, y	73.77 (5.31)	59.64 (2.59)	-24.155	<0.001^a, ***^
Female, n (%)	103	44	0.926	0.336^b^
Education, y	11.10 (4.99)	13.25 (4.12)	3.577	<0.001^a, ***^
MMSE	26.50 (2.53)	27.84 (2.12)	4.393	<0.001^a, ***^
APOE4 positivity, n (%)	38	14	0.906	0.341^b^
Clinical diagnosis, CN, n (%)	191 (100.00)	92 (100.00)		
Cerebral Aβ deposition				
Aβ retention, SUVR				
Baseline (n = 191, 92)	1.23 (0.24)	1.12 (0.14)	-4.783	<0.001^a, ***^
2-year follow-up (n = 137, 70)	1.27 (0.32)	1.11 (0.18)	-4.535	<0.001^a, ***^

Aβ=beta-amyloid, APOE4=apolipoprotein ε4, CN cognitively normal, AD Alzheimer’s disease, SUVR standardized uptake value ratio. Unless otherwise indicated, data are expressed as mean (standard deviation).

a by t-test

b by chi-square test.

* *p* < 0.05,

** *p* < 0.01,

*** *p* < 0.001.

Unlike the association between adiponectin level and Aβ deposition, adiponectin did not show any significant association with tau deposition, AD-signature neurodegeneration, and WMH volume. By contrast, a report from MCSA [23] showed that higher adiponectin level was associated with reduced cerebral glucose uptake and smaller hippocampal volume. This discrepancy may be explained by differences in the characteristics of study participants. While we targeted only CN individuals, the MCSA study included MCI patients as (n = 150) and CN individuals (n = 385). Inclusion of both cognitively impaired individuals and CN may have increased the variation in neurodegenerative and other brain pathologies in the previous study, thereby making it easy to detect the correlations with adiponectin level, compared to the current study, which included only CN individuals with relatively good brain condition. In addition, relatively small sample size (initial tau PET imaging, n= 73; 2-year follow-up tau PET imaging, n = 43) may also contribute to the lack of an observed relationship between adiponectin level and tau deposition.

The positive relationship between serum adiponectin and brain amyloid deposition demonstrated through both cross-sectional and longitudinal analyses is a novel finding. In addition, the present study had a couple of strengths. First, it included a relatively large sample of participants, who underwent comprehensive clinical assessments, blood tests and multimodal brain imaging to detect *in vivo* AD pathologies and cerebrovascular injury. Second, we comprehensively controlled potential confounders to investigate the association between serum adiponectin level and AD brain pathologies as clearly as possible. The study results were not affected by controlling for potential confounders. Nevertheless, our study had several limitations. First, the 2-year follow-up period for longitudinal analysis may not be adequate to detect the change of brain pathologies, such as tau deposition, AD-signature neurodegeneration, and WMHs, especially in CN individuals. This may contribute to the lack of observed association between adiponectin level and brain pathologies other than Aβ deposition. Further studies with a longer follow-up period are necessary to confirm these findings. Second, the initial tau PET was performed at an average of 2.5 years (standard deviation 0.3 years) after baseline adiponectin measurement, whereas the initial amyloid PET and MRI scans were performed at baseline. This temporal gap may have influenced our results. However, the results did not change after controlling for the temporal gap as an additional covariate. In addition, as mentioned previously, only a subset of study participants received tau PET scans, which made it difficult to detect any potential association between adiponectin level and tau deposition.

**Table 8:** Results of cross-sectional and longitudinal analyses of the associations between serum adiponectin and global Aβ retention in cognitively normal individuals according to age.

	Global Aβ retention, SUVR		Δ global Aβ retention, SUVR	
	β	*p-value*	β	*p-value*
Independent variable: adiponectin under high age (≥65 years) (n = 191, 137)				
Model 1^a^	0.292	<0.001^***^	0.231	0.016 ^*^
Model 2^b^	0.305	<0.001^***^	0.240	0.013 ^*^
Model 3^c^	0.302	0.003^**^	0.327	0.007^**^
Independent variable: adiponectin under low age (< 65 years) (n = 92, 70)				
Model 1^a^	-0.107	0.398	-0.170	0.228
Model 2^b^	-0.153	0.234	-0.164	0.267
Model 3^c^	-0.141	0.444	-0.249	0.238

Aβ=beta-amyloid, =standardized beta coefficients.

a Not adjusted.

b Adjusted for sex, and APOE4.

c Adjusted for sex, APOE 4, education, GDS score, VRS, BMI, annual income, LPA, albumin, fasting glucose, HDL- and LDL-cholesterol, HOMA-IR, zinc, alcohol intake, and smoking.

* *p* < 0.05,

** *p* < 0.01,

*** *p* < 0.001.

## Conclusions

The present findings from CN older adults suggest that blood adiponectin level is associated with increased brain amyloid deposition. Although further evidence is needed, the findings also indicate that adiponectin and the related signaling pathway may be a potential target for therapeutic and preventive strategies for AD.

## Data Availability

The data of the current study are not freely accessible because the IRB of the Seoul National University Hospital prevents public sharing of such data for privacy restrictions. However, the data can be available from the independent data sharing committee of the KBASE research group on reasonable request after approval by the IRB. Requests for data access can be submitted to the administrative coordinator of the KBASE group by e-mail (kbasecohort@gmail.com), who is independent of the authors.
